# Synthesis and Characterization of Vincristine and ε‑Viniferin-Loaded Folate-Conjugated PLGA–PEG Nanoparticles and Their Cytotoxic and Apoptotic Effects on Liver Cancer Cells

**DOI:** 10.1021/acsomega.5c10848

**Published:** 2026-04-23

**Authors:** Yüksel Öğünç Keçeci, Müzeyyen Demirel, Zerrin Seller

**Affiliations:** † Department of Biochemistry, Faculty of Pharmacy, 52944 Anadolu University, Tepebaşı, Eskişehir 26120, Türkiye; ‡ Department of Pharmaceutical Technology, Faculty of Pharmacy, Anadolu University, Tepebaşı, Eskişehir 26120, Türkiye

## Abstract

Recent studies highlight the potential of nanoparticles to enhance in vivo drug efficacy. Polylactide-*co*-glycolide acid (PLGA) is widely used for nanoparticle fabrication due to its biocompatibility, and PEGylation further improves its circulation time by reducing rapid reticuloendothelial system (RES) clearance. Folate-modified nanoparticles can additionally enable active targeting, as many cancer cells overexpress folate receptors. In this study, dual-drug-loaded and folate-targeted PLGA–polyethylene glycol (PEG) nanoparticles were developed, and their enhancing effects on cellular uptake, cytotoxicity, and apoptotic response were evaluated. The nanoparticles were prepared via the nanoprecipitation method. They exhibited a particle size of 276 ± 6 nm, a polydispersity index (PDI) of 0.5 ± 0.02, and a ζ potential of −19 ± 2 mV. The drug-loading capacity was 8.8% for ε-viniferine (EV) and 2.6% for vincristine sulfate (VS). The use of EV–VS-loaded PLGA–PEG-folate nanoparticles enhanced cytotoxicity and apoptosis compared to the free drug, reducing HepG2 cell viability from 94.3 to 53% at 10 μM EV + 1.64 μM VS and increasing early apoptotic cells from 15.3 to 31%, indicating their potential as a targeted drug delivery system for cancer cells.

## Graphical abstract

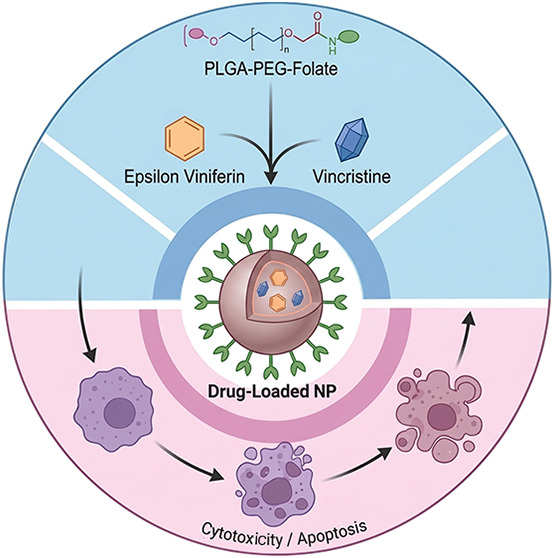

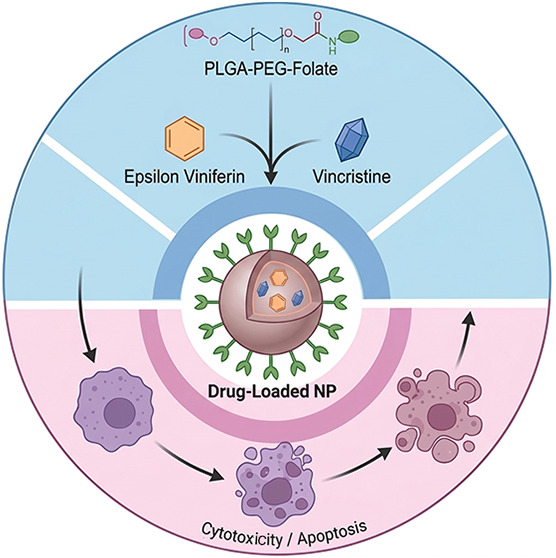

## 1 Introduction

The worldwide morbidity and mortality rates of cancer are rapidly increasing.^1,2^ Liver cancer is the sixth most common cancer. The statistics indicate that 8.3% of total global cancer deaths are composed of this particular type.^3^ Predictions suggest a rise of over 55% in the incidence of liver cancer cases and fatalities by 2040.^4^ Traditional cancer treatments such as radiotherapy, chemotherapy, surgery, and immunotherapy are used alone or in combination.^5–7^ However, limitations including nonselective toxicity, poor solubility, and inadequate targeting have driven the development of new strategies, particularly nanoparticle-based drug delivery systems.^8,9^

Very small polymeric nanoparticles, measuring 1–1000 nm, can be used to carry drugs. Formulating a therapeutic agent with polymeric nanoparticles can improve drug stability, solubility, and permeability, ultimately leading to enhanced therapeutic effects and decreased side effects.^10^ Polylactide-*co*-glycolide acid (PLGA) is a type of polymeric nanoparticle characterized by a lengthy half-life, minimal toxicity, and an inability to provoke an immune response.^11,12^ The biodegradable polymer PLGA has received approval from both the European Medicines Agency (EMA) and the US Food and Drug Administration (FDA) for its application in medical drug delivery systems.^13^ The components of PLGA, namely, glycolic acid and lactic acid, are readily metabolized within the Krebs cycle and are deemed safe from a toxicological standpoint.^14^

To deliver systemically administered drugs to target tissues at sufficient concentrations, they must circulate in the bloodstream for as long as possible.^15^ However, one limitation is their hydrophobic structure, which activates the reticuloendothelial system (RES) and leads to the removal of nanocarriers by opsonin proteins.^16^ Consequently, incorporation of a hydrophilic compound into the carrier system can prevent the elimination of nanocarriers by the reticuloendothelial system.^17^ PEG, which is a polyethylene glycol compound, is nontoxic and water-soluble and can be covalently bonded to proteins.^18^ Conjugation of polyethylene glycol (PEG) with PLGA nanoparticles (NPs) improves their pharmacological properties.^19^ Another effective method for enhancing biocompatibility, increasing stability, and evading biological systems is through the PEGylation of nanoparticles, which also increases stealthiness.^20^

Folic acid (FA) is widely used for targeted nanoparticle delivery because cancer cells overexpress folate receptors (FRs)—up to 500-fold higher than normal cells—facilitating selective uptake.^21^ Folate (vitamin B9) supports DNA synthesis and repair.^22^ FRs are essential for maintaining tumor proliferation and survival tumor.^23–25^ Tumor-associated folate receptors (FRα, FRβ, and FRγ) are ∼40 kDa glycoproteins, with FRα and FRβ being glycosylphosphatidylinositol (GPI)-anchored membrane forms, whereas FRγ is a soluble isoform.^26^

Vincristine (VS) is an alkaloid drug used as a chemotherapy drug.^27,28^ In 1963, vincristine, a vinca alkaloid, was sanctioned by the FDA for application in chemotherapy.^29^ It interacts with tubulin-expressing cells and triggers apoptosis.^30^ It is commercially marketed as “Oncovin”.^31,32^ It induces apoptosis by binding to tubulin and is commonly used in combination regimens, such as the MOPP protocol for Hodgkin’s lymphoma.^33^ Various types of cancer are treated using VS, including neuroblastoma, acute lymphocytic leukemia, acute myeloid leukemia, rhabdomyosarcoma, Wilms tumor, Hodgkin’s disease, and small cell lung cancer.^34,35^ It binds β-tubulin, blocking microtubule formation and thereby inhibiting cancer cell proliferation and spread.^36^ VS cannot distinguish between healthy and malignant cells and can attack any dividing cell without distinction.^37^ Multiple treatment cycles are necessary to ensure continuous exposure of malignant cells to VS. Inevitably, prolonged exposure to the drug at high doses can lead to adverse side effects.^38^ It has various side effects such as lung damage, peripheral neuropathy, a decrease in immune activity, headaches and sensory changes, hair loss, and constipation.^39,40^ Viniferin is a phenolic compound classified as a stilbenoid, also referred to as a phytoalexin. This molecule is a derivative of the compound that is the most researched in this group, resveratrol.^41^ ε-Viniferin (EV) is anti-inflammatory,^42^ antioxidant,^43^ antineoplastic,^44^ antiobesity^45^ and cardioprotective.^46^ It has been reported that the combination of vincristine sulfate and ε-viniferin at a lower concentration reduces cell growth and triggers apoptosis in HepG2 cells.^44^ While our previous work employed EV–VS-loaded PLGA–PEG carriers,^47^ there is no report of dual-drug loading of EV and VS in a folate-targeted PLGA–PEG system. In the current study, we synthesized and characterized EV–VS-loaded folate-conjugated PLGA–PEG nanoparticles (EV–VS PLGA–PEG-FA NPs) and evaluated their in vitro anticancer effects on HepG2 cells. The folate conjugation enables active targeting, while the dual-drug loading allows simultaneous delivery of EV and VS, potentially enhancing apoptotic pathways and optimizing drug release kinetics. By incorporating both drugs into a folate-targeted system, we aim to achieve higher efficacy than the free drug combination, leveraging both the known synergistic effects of EV and VS and the benefits of active targeting.

## 2 Materials and Methods

### 2.1 Materials

PLGA (*M*_w_ 7000 Da, LA/GA 50:50), PEG bisamine (*M*_w_ 3400 Da), folic acid, *N*-hydroxysuccinimide (NHS), dicyclohexylcarbodiimide (DCC) solution, dichloromethane (DCM), Dulbecco’s-modified Eagle’s medium (DMEM), vincristine sulfate, dimethyl sulfoxide (DMSO), and Tris–HCl were purchased from Sigma-Aldrich (St. Louis, MO). (3-(4,5-Dimethylthiazol-2-yl)-2,5-diphenyltetrazolium bromide) MTT and ε-viniferine were purchased from Applichem (Darmstadt, Germany). Trypsin–ethylenediaminetetraacetic acid (EDTA) and fetal bovine serum (FBS) were purchased from Biochrom in Berlin, Germany. The Annexin V-FITC apoptosis detection kit was obtained from BD in Franklin Lakes, New Jersey.

### 2.2 Methods

#### 2.2.1 Synthesis of PLGA–PEG-Folate Conjugate

The PLGA–PEG-folate conjugate was prepared in a 4-step process. The steps involved are the activation of folic acid, the activation of PLGA, the synthesis of folate-PEG-NH_2_, and the synthesis of the PLGA–PEG-folate conjugate.^48–50^

Step 1: Activation of folic acid: Folic acid was activated according to the method described by Patil et al., with slight modification.^49^ One gram of folic acid was dissolved in a mixture of 40 mL of anhydrous DMSO and 0.5 mL of triethylamine (TEA), and this solution was then stirred in the dark under a nitrogen atmosphere overnight. Following the addition of 0.5 g of dicyclohexylcarbodiimide and 0.28 g of *N*-hydroxysuccinamide (NHS), the mixture was stirred for a further 18 h. The byproduct dicyclohexyl urea (DCU) was eliminated through filtration. TEA and DMSO were removed by lyophilization under vacuum.

Step 2: Activation of PLGA polymer: Activation of PLGA was performed according to the method described by Zhao and Yung, with slight modification.^50^ About 0.4 g of PLGA was activated by mixing it with 0.01 g of DCC and 0.0062 g of NHS in 5 mL of dichloromethane at room temperature under a nitrogen atmosphere for 24 h. The mixture was then filtered to remove DCU. Activated PLGA was precipitated with cold diethyl ether after filtration, and then it was dried.

Step 3. Preparation of folic acid-PEG-NH_2_: Folic acid was conjugated to bisamine PEG (NH_2_–PEG–NH_2_) following the protocol of Patil et al., with minor modifications.^49^ About 0.4 g of bisamine–PEG (MW 3400 Da) was first dissolved in a mixture of 0.8 mL of acetonitrile, 0.4 mL of methylene chloride, and 32 mL of TEA, then the solution was mixed for 1 min. Subsequently, 0.1 g of NHS–folic acid was added, and the mixture was stirred in the dark overnight under a nitrogen atmosphere. The reaction was halted by the gradual addition of cold diethyl ether to precipitate the polymer and isolate the unreacted PEG. The filtered polymer was washed with diethyl ether after being precipitated. The unreacted polymer was removed through dialysis with a dialysis membrane (MWCO: 4000). The product was freeze-dried.

Step 4: Synthesis of PLGA–PEG-folic acid conjugate copolymer: The final synthesis was performed following the procedure reported by Zhao and Yung with slight modification.^50^ A mixture of 0.1 g of activated PLGA, 0.12 g of folate-PEG-NH_2_, and 5 mL of DMSO was combined at room temperature in a nitrogen atmosphere for 8 h. The product was precipitated with cold diethyl ether and subsequently dissolved in DMSO. Dialysis of the sample was carried out against deionized water employing a dialysis membrane (MWCO: 12,000) for a period of 72 h to remove DMSO and any unreacted polymers. The collected copolymer (folate-PEG–PLGA) was dialyzed in DMSO using a dialysis membrane (MWCO: 14,000) to eliminate the PLGA–PEG–PLGA copolymer. The dialyzed copolymer was subsequently recovered after dialyzing against deionized water for 72 h and then freeze-dried using a Scanvac device, which is manufactured in Denmark.

Spectra of Fourier transform infrared (FTIR) and NMR were obtained. ^1^H NMR spectra were recorded using a Bruker Ascend 300 MHz digital FT-NMR spectrometer in CDCl_3_ solvent. The ^1^H NMR measurement conditions were as follows: sample concentration approximately 2%, measurements performed at ambient temperature, and 179 scans acquired for each spectrum. IR spectra of the solid samples were obtained using a Shimadzu IRAffinity-1S spectrophotometer. The IR measurement conditions were: spectral range of 4000–400 cm^–1^, 10 scans per spectrum, a spectral resolution of 4 cm^–1^, and an ATR accessory (PIKE MIRacle).

#### 2.2.2 Preparation of EV–VS-Loaded PLGA–PEG-Folate Nanoparticles

Fifteen mg of PLGA–PEG-folate was dissolved in a mixture of 1.3 mL of DCM and 0.65 mL of acetone. EV (0.25 mg/mL, ethanol) and VS (0.3 mg/mL, distilled water) were dissolved separately, and then an equal amount of both compounds was transferred into a polymer solution. The mixture was subjected to sonication for a duration of 30 s at an amplitude of 20%. The solution was then added to 7.5 mL of 0.1% pluronic F-68. The mixture was left to sit at room temperature for 45 min. Following this step, DCM and acetone were removed using a rotary evaporator at 30 °C with 50 rpm.

#### 2.2.3 Quantification of Drug Encapsulation

The amount of drug encapsulated in nanoparticles was determined by dissolving accurately weighed samples in a mobile phase made up of methanol and a triethylamine solution, which had a pH of 7.5 and a 70:30 v/v ratio.^51^ The solution was then diluted and examined using high-performance liquid chromatography (HPLC), a Shimadzu LC-20AT model (automatic injection and photodiode array (PDA) detector) from Japan. Analyses were performed by a C18 column (column diameter: 4.6 mm, column length: 25.0 cm, and particle diameter: 5 μm) at 297 nm wavelength with a flow rate of 1 mL/min. The experiment was repeated three times. The standard curves were as follows: EV: *y* = 29,340.98*x* + 1916.49, VS: *y* = 8359.98*x* – 642.76. The drug-loading percentage (DL%) was determined by eq 1.^52^ No aggregation was observed in relation to the drug content.

1 DL%=(massofdruginnanoparticle/massofnanoparticlerecovered)×100

#### 2.2.4 Evaluation of Particle Size, ζ-Potential, and Polydispersity Index

The particle size, surface charge (ζ potential), and size distribution (polydispersity index) of drug-loaded nanoparticles were studied with a Malvern Zetasizer Nano-ZS (Malvern, U.K.). Particle size and zeta potential measurements were performed at 25 °C using filtered distilled water. Particle size (dynamic light scattering, DLS) measurement conditions were as follows: scattering angle of 90°, refractive index of 1.34, and absorbance of 0.01. ζ potential measurements were performed with sodium chloride added to regulate the conductivity to 50 μS/cm.

#### 2.2.5 Assessment of In Vitro Drug Release and Release Kinetics

Nanoparticle release kinetics were studied using the dialysis approach. A 25 μL aliquot of drug-loaded nanoparticles, composed of 0.2% w/v PLGA-*b*-PEG, 0.01% w/v EV, and 0.01% w/v VS, was placed in a dialysis bag (MWCO: 14,000 Da) and immersed in 6 mL of Tris–HCl buffer (pH 7.4). The release system was maintained at 37 °C (±0.5) with stirring at 100 rpm (±2). At designated intervals (5, 15, 30 min; 1, 3, 4, 6, 12, 24 h), 30 μL samples were withdrawn from the outer medium, and the same amount of fresh medium fluid was added to maintain sink conditions. The samples were analyzed using HPLC. Each experiment was performed in triplicate, and parallel measurements were conducted with free drug solutions (*n* = 3).^53^ The standard curves were as follows: EV: *y* = 44,586.4*x* + 839.93, VS: *y* = 8274.12*x* – 1636.69.

In vitro release profiles of EV and VS from PLGA–PEG-FA NPs in Tris–HCl buffer (pH 7.4) were applied to different kinetic models (zero-order kinetics, first-order kinetics, Higuchi model, Korsmeyer–Peppas model, Hixson–Crowell model, and Hopfenberg model).

#### 2.2.6 Characterization of Thermal Properties

Differential scanning calorimetry (DSC-60, Shimadzu, Kyoto, Japan) was employed to investigate the thermal behavior of pure drugs, the polymer alone, the physical mixture (PM) of EV, VS, and PLGA–PEG-folate, and lyophilized nanoparticles containing the drug. Analyses were carried out using aluminum sample pans in the 30–300 °C range, with a 10 °C/min heating rate, under nitrogen at 50 mL/min.

#### 2.2.7 Cell Culture

HepG2 cells (ATCC in Manassas, VA) were cultured in Dulbecco’s-Modified Eagle Medium (DMEM) supplemented with 10% fetal bovine serum (FBS), 3.7 g/L NaHCO_3_, and 1% penicillin–streptomycin solution, within an incubator set at 5% CO_2_ and 37 °C. Cells were treated with a trypsin/EDTA solution at intervals of 2–3 days and then subcultured in a 1:3 ratio.

#### 2.2.8 Measurement of Cytotoxicity

Viability of cells was assessed using the 3-(4,5-dimethylthiazol-2-yl)-2,5-dimethyl tetrazolium bromide (MTT) test.^54^ In total, 1 × 10^5^ cells were added to 96-well plates and were incubated at 37 °C for 24 h. Various combinations of EV- and VS-loaded nanoparticles and free drugs at different concentrations were added to each well, after which the plates were incubated for a further 24 h. At the end of this period, the cells were incubated with MTT to a final concentration of 0.5 mg/mL for an additional 4 h at 37 °C. Following incubation, the culture medium was removed, and 200 μL of dimethyl sulfoxide (DMSO) was added to dissolve the formazan crystal. The plates were subjected to gentle shaking for 10 min at room temperature. The microplate system (ELx800 BioTek) was used to measure absorbance at a wavelength of 540 nm. Experiment was conducted three times.

#### 2.2.9 Detection of Early Apoptosis by Flow Cytometry

Apoptotic cell detection was performed using the “FITC Annexin V Apoptosis Detection Kit II (BD Pharmingen)” in accordance with the manufacturer’s guidelines. HepG2 cells (3 × 10^5^ cells/well) were incubated for 12 h after treatment with either free drugs or the combined use of EV and VS nanoparticles. Each tube then received 100 μL of binding buffer after being washed twice with cold phosphate-buffered saline (PBS). 5 μL of FITC Annexin V and 5 μL of propidium iodide dye were added to each tube, except for the negative control cells, and the tubes were then incubated in the dark for 15 min at room temperature. Following a 15 min interval, an additional 400 μL of binding buffer was added to the tubes and then examined on a flow cytometer, specifically the Becton-Dickinson FACS Aria model from the USA.

#### 2.2.10 Statistical Analysis

Data analyses were performed using GraphPad Prism 8.0 software. Differences between groups were evaluated by one-way analysis of variance (ANOVA) followed by Tukey’s post hoc tests for multiple comparisons. A *p*-value of <0.05 was considered statistically significant for differences.

## 3 Results and Discussion

### 3.1 Synthesis and Characterization of EV–VS-Loaded Folate-Conjugated PLGA–PEG Nanoparticles

The nanoprecipitation method is increasingly preferred in pharmaceutical research due to its simplicity, scalability, reproducibility, minimal use of toxic solvents, and ability to produce uniform submicrometer particles with low energy input.^55^ Nanoprecipitation was demonstrated to be a suitable alternative for the encapsulation of natural compounds such as EV and VS. In this research, EV–VS PLGA–PEG-FA NPs were successfully synthesized using the nanoprecipitation method with a PLGA–PEG-folate conjugate and a drug solution. The PLGA–PEG-folate conjugate was synthesized through a four-step process. The steps involved are the activation of folic acid, the activation of PLGA, the synthesis of folate-PEG-NH_2_, and the synthesis of the PLGA–PEG-folate conjugate. As a result of the migration of water-miscible acetone and DCM into the water phase, nanoparticles of PLGA spontaneously form through precipitation. We created a PLGA–PEG-FA conjugate and confirmed its structure through proton nuclear magnetic resonance (^1^H NMR) and Fourier transform infrared (FTIR) spectroscopy, as shown in Figure 1.

**Figure 1:**
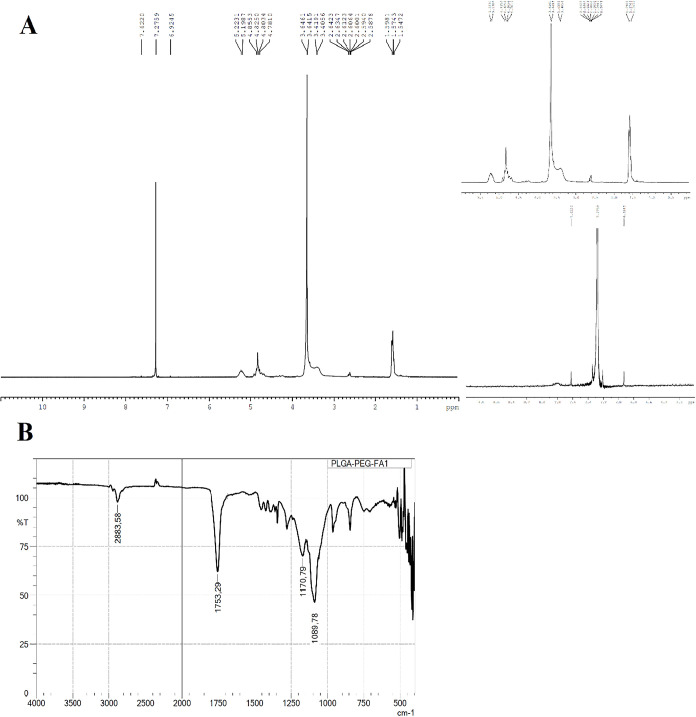
Characterization of PLGA–PEG-FA conjugate. (A) ^1^H NMR spectra of PLGA–PEG-FA. (B) FTIR spectrum of PLGA–PEG-FA, showing the characteristic functional group peaks.

FTIR and ^1^H NMR are powerful tools for investigating interaction possibilities between formulation components and active agents. The most commonly examined active nucleus in NMR is ^1^H. Because of differences in chemical shift, NMR signals can be assigned to specific molecules or their segments. Simple ^1^H spectroscopy enables rapid and straightforward detection of ionic interactions between components. Therefore, FTIR and ^1^H NMR spectroscopic analyses were performed.^56,57^

In the ^1^H NMR spectrum, distinct signals for PEG (−CH_2_CH_2_O– at 3.64 ppm) and PLGA (methine and methyl protons of lactide at 5.2 and 1.6 ppm, glycolide methylenes at 4.8–4.3 ppm) were easily visible. The conjugate also showed multiple resonances in the 6.5–8.2 ppm range, which are attributed to the aromatic protons of folic acid. The peaks were not visible in the spectra of the untouched PLGA–PEG, indicating that the folic acid conjugation was successful. Fourier transform infrared analysis further supported the structure. Verification of the PLGA and PEG backbones was confirmed by strong absorptions at 1753 cm^–1^ (ester C=O stretch) and 1170–1089 cm^–1^ (C–O–C ether vibrations). Despite being weaker due to lower molar incorporation, the presence of folic acid-related signals was consistent with covalent attachment. The results collectively validate the effective synthesis of PLGA–PEG-FA. The inclusion of folic acid serves as a targeting component for potential drug delivery systems, facilitating a selective uptake by tumor cells expressing the folate receptor.

### 3.2 Drug Loading

Drug loading depends on drug solubility in the excipient matrix, influenced by matrix composition, molecular weight, drug–polymer interactions, and functional groups. PEG is often preferred as it minimally affects loading and interactions.^58^ Despite their benefits, nanoparticles have limitations: small size and large surface area can cause low drug loading and burst release.^59^ The drug-loading percentages were 8.8 ± 0.6 and 2.6 ± 0.2 for EV and VS, respectively, as determined by HPLC analysis mentioned earlier. The data in question are comparable to those found in early studies.^60^ EV is sparingly soluble in water, whereas VS is freely soluble in water. This study has demonstrated that varying drug-loading percentages of EV and VS are associated with their respective polar characteristics. The hydrophobic properties of PLGA led to the low encapsulation efficiency of these anticancer agents.

### 3.3 Particle Size, PDI, and ζ Potential

Nanoparticle size and size distribution are crucial, as they determine in vivo distribution, fate, toxicity, targeting, drug loading, release rate, and stability.^58^ The data represent an average of 10 batches and 3 repeats. The data are an average of 10 batches and 3 repeats.

The particles between 100 and 200 nm generally show prolonged circulation and reduced clearance, making this size range optimal for effective colloidal drug delivery systems.^61^ The particle size of EV–VS PLGA–PEG-FA NPs was found to be 276 ± 6 nm. Figure 2 illustrates the particle size distribution of the nanoparticles by dynamic light scattering (DLS). The enhanced permeability and retention (EPR) effect was first described over 2 decades ago and has since become a key principle in the design of nanoparticle-based systems for the detection and treatment of solid tumors. Tumor vasculature is highly permeable due to enlarged interendothelial gaps, which can reach several hundred nanometers, in contrast to the <10 nm gaps observed in normal vessels.^62^ Passive accumulation of nanoparticles via the EPR effect is therefore a central strategy in cancer nanomedicine. However, the optimal nanoparticle size required to overcome physiological barriers—such as glomerular filtration or vascular fenestration—remains incompletely understood, limiting the clinical translation of these systems.^63^ Size is a critical determinant of EPR-mediated delivery. Studies suggest that nanoparticles between 40 and 400 nm achieve prolonged circulation and enhanced tumor accumulation while minimizing renal clearance.^63,64^ A review of 297 studies published from 2005 to 2021 further indicated that, in certain cases, nanoparticles with hydrodynamic diameters above 200 nm exhibited superior tumor delivery efficiency compared to those in the 10–200 nm range.^65^ Moreover, functionalization with active targeting ligands, such as folate, can further enhance tumor-specific uptake beyond passive EPR-driven accumulation, enabling effective targeting even for particles larger than 200 nm.^66^ Collectively, these findings indicate that the prepared EV–VS PLGA–PEG-FA nanoparticles (∼276 nm) fall within a size range considered suitable for EPR-mediated tumor targeting.

**Figure 2:**
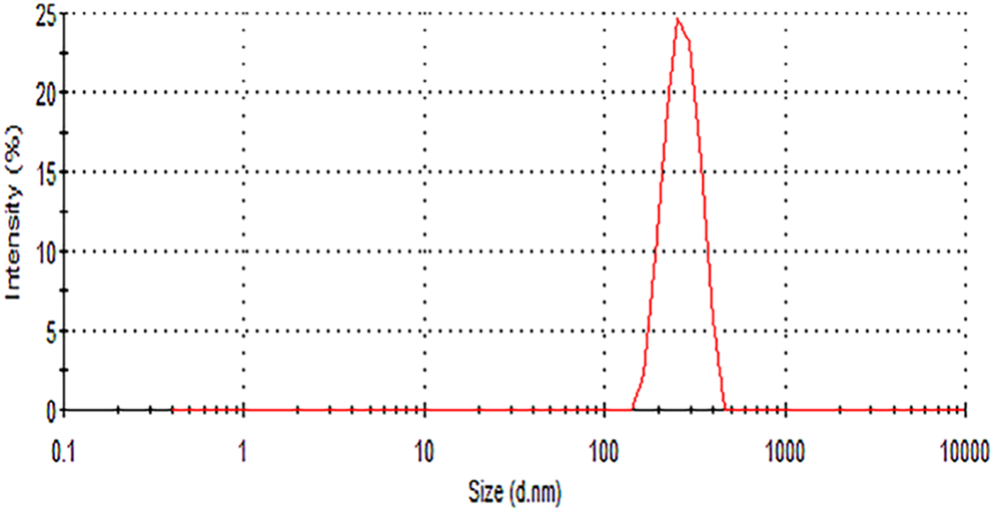
Particle size distribution of EV–VS PLGA–PEG-folate nanoparticles.

The particle size distribution index (PDI), or dispersity, describes the uniformity of particle sizes. The numerical value of PDI ranges from 0.0 (for a perfectly uniform sample with respect to the particle size) to 1.0 (for a highly polydisperse sample with multiple particle size populations). Values of 0.2 and below are most commonly deemed acceptable in practice for polymer-based nanoparticle materials.^67^ The PDI value of EV–VS PLGA–PEG-FA NPs is 0.57 ± 0.02, indicating a distribution was very broad and that aggregates and small particles coexisted. This is a limitation, and formulation parameters may require further optimization to achieve a narrower size distribution in future studies. Filtration is another effective strategy to reduce particle size heterogeneity. However, due to the limited total mass of the formulation and to prevent potential loss of yield, a filtration step was not implemented in the present study.

According to literature (Esmaeili et al.), the ζ potential value of the PLGA–PEG polymer was −12.2 ± 0.6 mV. In this study, EV–VS PLGA–PEG-FA NPs displayed a negative ζ potential of −19 ± 2 mV in an alkaline solution (pH 7.2) due to the presence of terminal carboxylic groups in the polymer. The presence of EV and VS in the nanoparticles may have lowered the negative ζ potential by covering surface carboxyl groups via drug adsorption. Additionally, NH_2_ groups in the folic acid moiety contributed to a less negative potential in folate-modified nanoparticles.^60,68^

ζ potential is a key indicator of colloidal nanoparticle stability. Higher positive or negative values enhance repulsive forces between particles, reducing aggregation and improving stability.^69^ Nanoparticles with a ζ potential exceeding ±30 mV have been demonstrated to be stable in suspension, where the surface charge inhibits aggregation of the particles.^58^ The ζ potential (−19 mV) suggests moderate stability but is below the ±30 mV threshold usually required for colloidal stability. Pluronic F-68 has been widely and successfully used in nanomedical systems, and its effectiveness as a polymeric surfactant has been well demonstrated. Pluronic F-68 is a triblock polymer (PEO–PPO–PEO), which can form a hydrophobic core and a hydrophilic shell in aqueous media. This core–shell structure prevents direct contact and aggregation between particles, thereby providing steric stabilization.^70^ However, studies also show that this stabilization is not always automatically achieved. For example, in some PLGA nanoparticles, F-68 alone may not provide sufficient stability; therefore, system-specific parameters such as the type of polymer used, surface chemistry, and washing/processing steps are critical.^71^ These findings indicate that the EV–VS PLGA–PEG-FA NPs formulation could be suitable for cellular uptake, taking into account its small particle size.

Particle uptake is influenced by surface charge: high ζ-potential supports endocytic internalization, and cationic or neutral nanoparticles are generally internalized more efficiently than anionic ones.^72^ The anionic charge of cells promotes the uptake of positively charged NPs, raising the toxicity risk for normal cells. Negatively charged nanoparticles are preferable for drug delivery, as they resist macrophage opsonization and plasma protein aggregation.^73^ The negative ζ-potential results of EV–VS PLGA–PEG-FA NPs indicate that the developed nanoparticles would be more suitable in this regard.

### 3.4 Differential Scanning Calorimetry

Differential scanning calorimetry (DSC) was used to assess the thermal properties and physical state of drugs within nanoparticles, as this influences drug release. In polymeric carriers, drugs may exist in amorphous or crystalline forms, or as solid solutions/dispersions within amorphous or crystalline polymers.^74^ Figure 3 shows the DSC thermogram of VS, EV, PLGA-*b*-PEG, FA, PM, and NPs up to 300 °C. DSC analysis of pure VS showed no obvious melting peak and exhibited thermal decomposition behavior starting at approximately 240 °C. The DSC curve of pure EV showed a broad endothermic behavior (210–300 °C) followed by thermal decomposition.^47^ The degree of crystallinity in PLGAs can range from completely amorphous to completely crystalline, depending on their block structure and molar ratio. PLGAs generally exhibit a glass transition temperature over a range of 40–60 °C. The thermal behavior of PLGA-*b*-PEG exhibited two distinct features: the first, appearing between 40 and 60 °C, corresponds to the glass transition temperature of the PLGA segment; the second, a broad peak around 275 °C, is associated with the onset of PEG degradation (Figure 3). At room temperature, solid folic acid is crystalline in form.^75^ Despite having no observed melting point, “However” appears to rapidly degrade and decompose through an endothermic reaction at approximately 180 °C. The PM thermogram closely resembled that of the pure polymer. Conversely, the DSC thermogram of NPs displayed no thermal peaks, but it did decompose after 270 °C.

**Figure 3:**
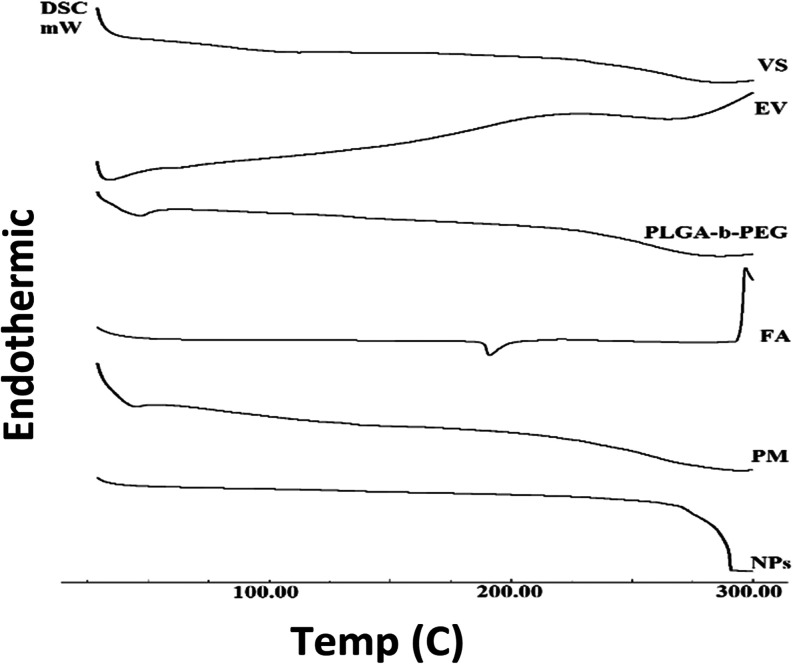
Thermal behavior of free substance (EV or VS), PLGA-*b*-PEG, physical mixture, and nanoparticles. EV: ε-viniferin, VS: vincristine sulfate, PLGA-*b*-PEG: poly­(ethylene glycol) methyl ether-*block*-poly­(lactide-*co*-glycolide), FA: folic acid, PM: physical mixture of three substances (EV, VS, and folate-conjugated PLGA–PEG), and NPs: EV/VS-loaded nanoparticles.

### 3.5 In Vitro Drug Release and Release Kinetics

Nanoparticles can enhance drug delivery and potency but may also increase carrier-related toxicity, making quality and performance assessment crucial. In vitro drug release is essential for evaluating the safety and efficacy of nanoparticulate formulations.^76^ In vitro release studies of the prepared NPs were conducted.

The rollout of pure EVs and pure VS vehicles was significantly behind that of the NPs. The release of pure EV in vitro was 5.6% at the end of 4 h and reached 66.5% for VS; meanwhile, the in vitro release of pure EV increased to 28.2 and 91.4% when formulated with EV–VS PLGA–PEG-FA NPs. The poor water solubility of EV and the amorphous structure of NPs may be responsible for this outcome.

In general, in vitro drug release from nanoparticles synthesized by nanoprecipitation encompasses two phases: an initial phase of “burst release”, which is succeeded by a subsequent phase of prolonged release.^55,77^ The in vitro release profiles of the NPs were studied in Tris–HCl solution at a pH of 7.4 using a dialysis membrane method at a temperature of 37 ± 0.5 °C. Folate-modified PLGA–PEG NPs show a biphasic release profile: an initial rapid release is followed by a steady and continuous release pattern (Figure 4). Initial release rates were rapid in the first 1 h at 14.2 ± 3.1 and 30.3 ± 2.9% for EV and VS, respectively, from the NPs. Within the time span of 1–24 h, a consistent release rate was maintained. Within 24 h, accumulative release amounts of 30.0 ± 6.0 and 100 ± 5.2% were observed for EV and VS, respectively, from PLGA–PEG-FA nanoparticles. The initial burst may be linked to the rapid discharge of drugs absorbed and loosely bound to the high surface area of the nanoparticles and in the water channels within the nanoparticles, while the steady release is likely due to the sustained release of encapsulated vesicles and vesicle structures following the gradual degradation of nanoparticles and/or slow diffusion through the polymer matrix of the drug localized in the PLGA core of the nanoparticles.^19,78,79^

**Figure 4:**
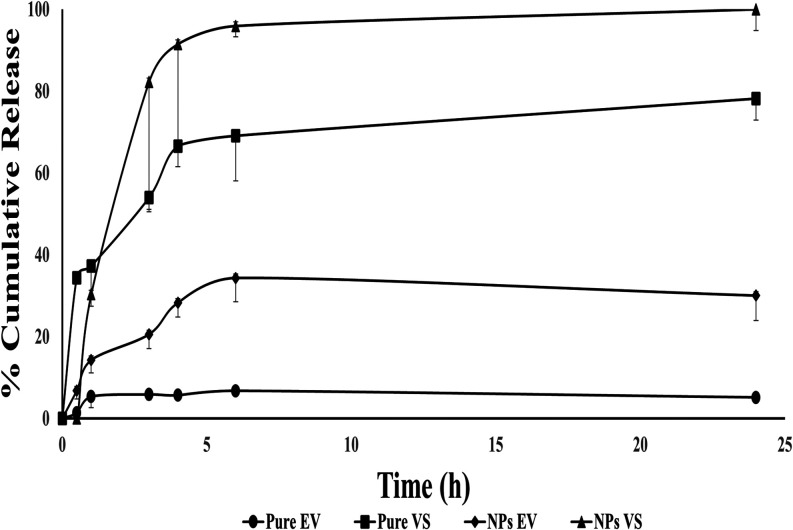
In vitro release profiles of EV and VS from free substances and EV/VS-loaded PLGA–PEG-folate nanoparticles. EV: ε-viniferin, VS: vincristine sulfate, and NPs: nanoparticles (*n* = 3).

Figure 4 illustrates a biphasic release of active agents from nanoparticles with levels significantly higher than those seen with pure EV and pure VS. The probable release of EVs and VSs from NPs could be caused by a decrease in the crystallinity of active pharmaceutical ingredients (APIs) during the lyophilization process of NP preparation methods.

The release data were investigated by *k*, *r* ^2^, AIC, and MSC for the selection of the best fitted kinetic model, and the Korsmeyer–Peppas model was the best fitted model for pure EV, pure VS, and NP EV according to the highest *k*, *r* ^2^, and MSC values with the lowest AIC data (Table 1). The first-order kinetic model was fitted for NPs VS according to the highest *r* ^2^ and MSC values, while the Korsmeyer–Peppas model was fitted for NPs VS according to the highest *k* and lowest AIC data. The first-order release model showed that the release of the VS depends on the concentration of the drug. The Korsmeyer–Peppas model was the best model that the release data of pure EV, pure VS, and NPs EV were fitted, which indicates that the drug release is ruled by both diffusion of the drug as well as the dissolution/erosion of the polymeric matrix.^80^

**Table 1:** Kinetic Modeling of EV and VS Release from Pure EV and VS and PLGA–PEG-FA NPs by DDsolver Software Program^a^

		kinetic model					
code	criteria	zero order	first order	Higuchi	Korsmeyer–Peppas	Hixon–Crowell	Hopfenberg
pure EV	*k*	0.3296	0.0034	1.8065	**3.3112**	0.0011	0.0011
	*r* ^2^	–1.5433	–1.5066	–0.1036	**0.4469**	–1.5187	–2.0224
	AIC	34.1971	34.0952	28.3526	**24.2415**	34.1290	36.1290
	MSC	–1.8613	–1.8468	–1.0264	**–0.4391**	–1.8516	–2.1373
	*n*				0.2753		3.0000
pure VS	*k*	4.3455	0.1198	21.8295	**45.1711**	0.0320	0.0320
	*r* ^2^	–1.0163	0.2052	0.4343	**0.9367**	–0.0187	–0.2225
	AIC	65.5364	59.0198	56.6394	**42.0280**	60.7574	62.7574
	MSC	–1.7679	–0.8370	–0.4969	**1.5904**	–1.0852	–1.3709
	*n*				0.1750		3.0000
NPs EV	*k*	1.7535	0.0313	8.8958	**11.7270**	0.0066	0.0066
	*r* ^2^	–0.6294	–0.4088	0.4811	**0.9797**	–0.4964	–0.7957
	AIC	53.6032	52.5846	45.5931	**19.6862**	53.0070	55.0070
	MSC	–1.2474	–1.1018	–0.1031	**3.0049**	–1.1622	–1.4479
	*n*				0.6064		3.0000
NPs VS	*k*	5.6717	0.5582	28.4378	**32.8950**	0.0743	0.0743
	*r* ^2^	–0.1890	**0.9393**	0.6024	0.8919	0.6594	0.8148
	AIC	69.1882	48.3696	61.5200	**44.7255**	60.4367	56.8950
	MSC	–1.2071	**1.7670**	–0.1116	1.1125	0.0432	0.5491
	*n*				0.6850		3.0000

a The data in bold font show the selected release kinetic models.

### 3.6 In Vitro Cytotoxicity Assay

The impact of EV–VS PLGA–PEG-FA nanoparticles and free EV–VS on HepG2 cell viability was assessed at the same drug dosages (20 μM EV + 3.28 μM VS; 10 μM EV + 1.64 μM VS; and 5 μM EV + 0.82 μM VS), and cell viability results are presented in Figure 5. At the same drug concentration, nanoparticle formulations exhibit greater cytotoxicity than free drugs. Following treatment of HepG2 cells with EV–VS PLGA–PEG-FA NPs at a concentration of 10 μM EV and 1.64 μM VS, the cell viability was 53.9%. In contrast, treatment with free EV–VS at the same concentration resulted in a cell viability of 94.3%. Patra et al. discovered that the IC_50_ value was 51.48 mg/mL for free genistein; however, they found it to be 11.98 mg/mL for genistein-loaded PLGA–PEG-folate nanoparticles when tested on SKOV-3 cells.^60^ The findings suggest that the use of the folic acid receptor-targeting PLGA–PEG nanoparticle carrier system with the active substance demonstrated enhanced anticancer effects compared to its use without the carrier system.

**Figure 5:**
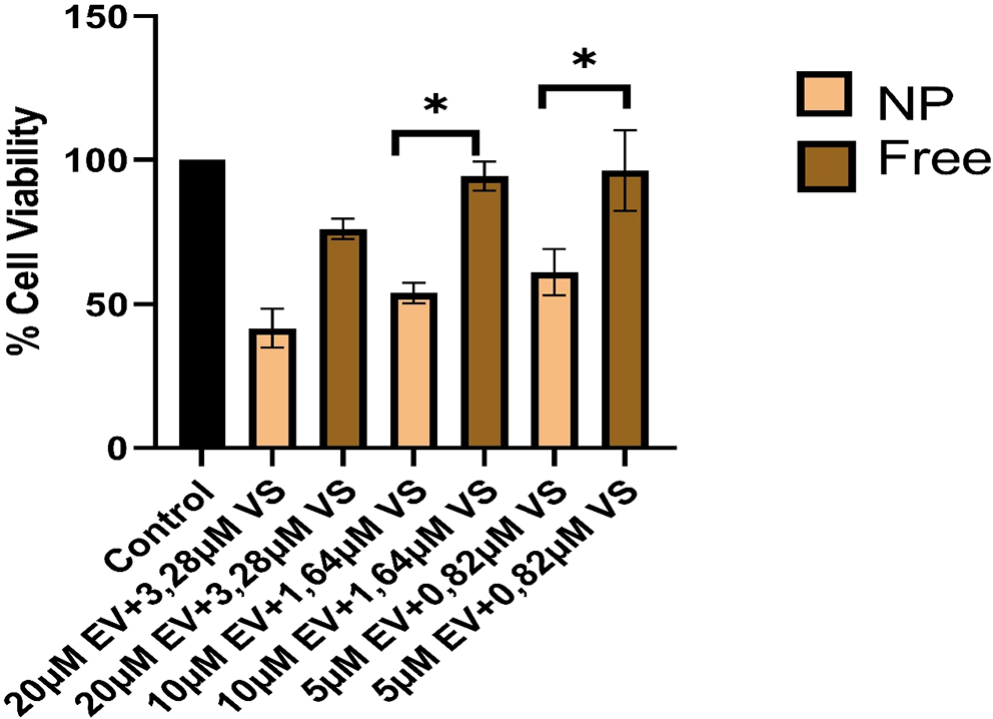
Cytotoxic effects of free and EV–VS-loaded PLGA–PEG-folate nanoparticles (NP) (*n* = 3).

### 3.7 Detection of Apoptotic Cells

The early apoptotic cell percentages in HepG2 cells, which had been treated with a concentration of 10 μM EV and 1.64 μM VS, were found to be 15.3 and 31% for the free and nanoparticle formulations, respectively. When cells were exposed to a concentration of 5 μM EV combined with 0.82 μM VS, it was found that the early apoptotic cell percentages were 11.3% for the free formulation and 27.3% for the nanoparticle formulation (Figure 6). At a similar concentration, the folate receptor-targeted nanoparticles were found to induce apoptosis more effectively than EV–VS by itself. The study conducted by Pillai et al. demonstrated that the use of the PLGA–PEG-folate nanoparticle system to deliver curcumin enhanced its capacity to trigger apoptosis.^81^

**Figure 6:**
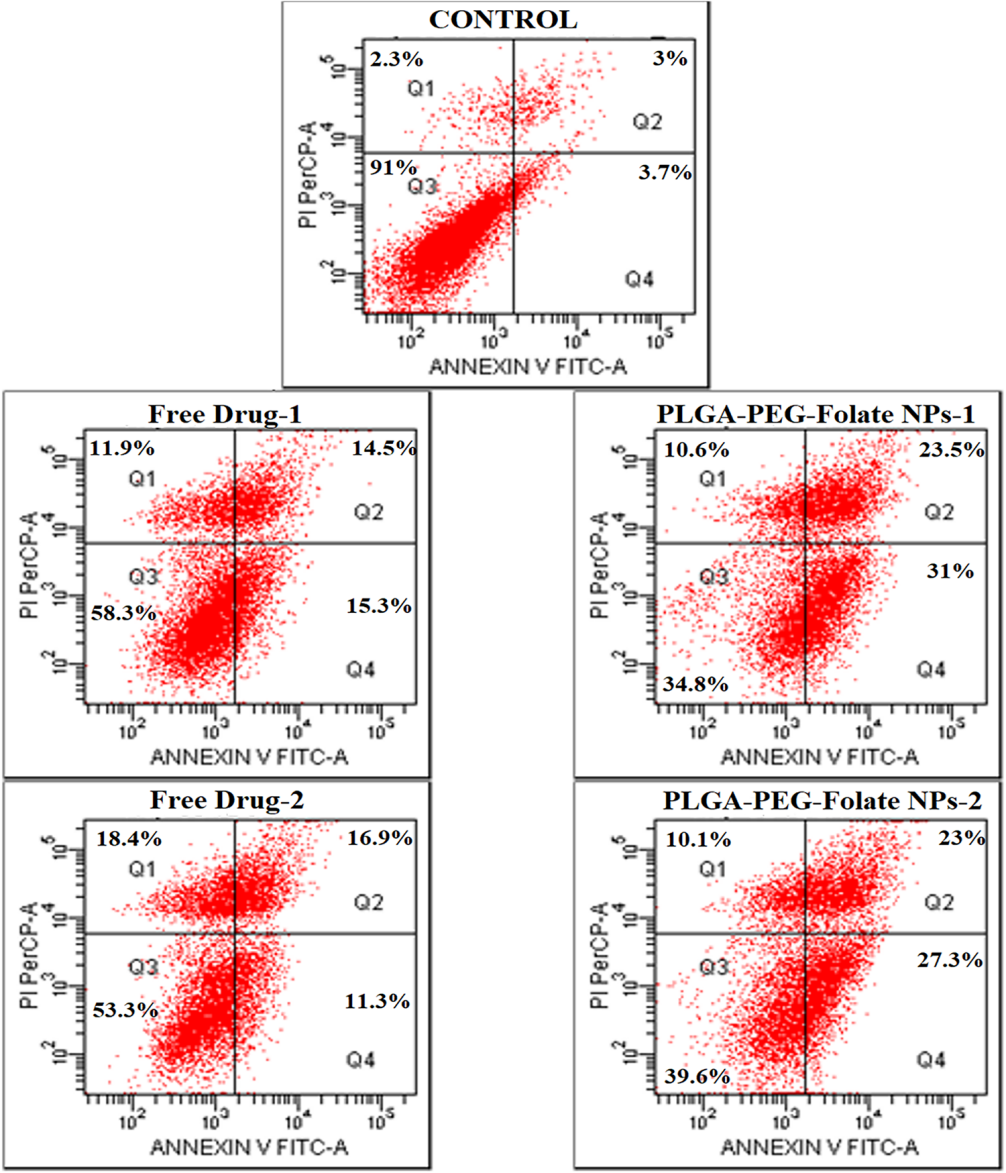
Apoptotic percentage of free combination of EV/VS drugs and EV/VS-loaded PLGA–PEG-folate nanoparticles on HepG2 cells. Free Drug-1 and PLGA–PEG-folate NPs-1: 10 μM EV/1.64 μM VS; Free Drug-2 and PLGA–PEG-folate NPs-2: 5 μM EV/0.82 μM VS (Q1: Necrotic cells, Q2: late apoptotic cells, Q3: viable cells, and Q4: early apoptotic cells) (*n* = 1).

A major mechanism underlying the enhanced uptake of folate-targeted nanoparticle systems (e.g., folate-conjugated PLGA–PEG nanoparticles) by cancer cells is receptor-mediated endocytosis. FRs are typically expressed at low levels in most normal cells, whereas they are overexpressed in many tumor cells; this differential expression enables tumor-specific uptake of folate-conjugated nanoparticles.^82^ Through this mechanism, numerous studies have demonstrated that folate-conjugated nanocarriers achieve more efficient intracellular drug accumulation compared to nonfolate formulations. For example, folate-modified PLGA nanoparticles showed significantly increased internalization in HeLa cells.^83^

In addition, targeted uptake not only facilitates cellular entry of nanoparticles but also enables deeper subcellular interactions. For instance, in vitro and in vivo studies on doxorubicin (DOX)-loaded folate-terminated polyrotaxane nanoparticles have shown specific accumulation in organelles such as mitochondria and the endoplasmic reticulum, thereby activating mitochondria-mediated apoptotic pathways. These systems were reported to induce mitochondrial membrane permeabilization, cytochrome-*c* release, subsequent caspase-9 and caspase-3 activation, suppression of the antiapoptotic protein Bcl-2, and upregulation of pro-apoptotic proteins such as Bax and Bid, ultimately triggering robust apoptotic cell death.^84^ Similarly, disulfiram-loaded folate-targeted PLGA–PEG nanoparticles have been shown to enhance reactive oxygen species (ROS) generation in both in vitro and in vivo models, leading to increased apoptosis and inhibition of cell proliferation.^85^ These findings suggest that the improved delivery of the encapsulated drug through folate receptor-mediated uptake can trigger ROS-mediated stress and apoptotic signaling within the cell. In light of this literature, the pronounced cytotoxic and pro-apoptotic effects observed with our EV–VS-loaded PLGA–PEG-FA nanoparticle system in HepG2 cells are likely attributable to these mechanisms.

When genistein-loaded PLGA–PEG-FA nanoparticles were applied against ovarian cancer cells (SKOV-3), the IC_50_ value decreased from 51.48 μg/mL for free genistein to 11.98 μg/mL for the targeted nanoparticle formulation, indicating approximately a 4–5-fold increase in cytotoxic potency.^60^ Similarly, curcumin-loaded PLA–PEG-Fol micelles exhibited enhanced cellular uptake and cytotoxicity in HepG2 cells compared to curcumin-loaded PLA–PEG micelles. This finding further demonstrates the critical role of folate in improving the targeting capability and cellular uptake of micellar systems.^86^ Overall, nanoparticle-mediated drug delivery—particularly when combined with folate targeting and PEGylation—consistently enhances anticancer efficacy relative to free drug forms.

Cytotoxicity and apoptosis analyses were performed only on HepG2 cancer cells without comparison to a nonmalignant hepatic cell line, thereby restricting conclusions regarding selective toxicity. Therefore, future studies should include healthy liver cell lines to better evaluate the cancer-specific effects.

## 4 Conclusions

Ongoing research continues to focus on developing novel anticancer agents and strategies that enhance efficacy while minimizing side effects. In this study, folate was used as a targeting ligand due to the overexpression of folate receptors in cancer cells, and the biocompatible PLGA–PEG carrier was loaded with EV, which exhibits synergistic effects when combined with the anticancer drug VS. EV–VS-loaded PLGA–PEG-FA nanoparticles were successfully synthesized and characterized, showing suitable size (276 ± 6 nm), uniform dispersion (PDI 0.5 ± 0.02), negative ζ potential (−19 ± 2 mV), and stable physical properties that favor cellular uptake. The nanoparticles provided controlled, two-phase release of EV and VS, enhancing bioavailability compared to the free drugs. Biologically, the targeted delivery system significantly increased cytotoxicity and induced greater early apoptosis in HepG2 cells, demonstrating the advantages of folate-mediated targeting and dual-drug loading. These findings suggest that EV–VS-loaded PLGA–PEG-FA nanoparticles are a promising platform for anticancer therapy, offering controlled drug release, targeted distribution, and enhanced therapeutic efficacy over free drug combinations. However, this study was conducted entirely in vitro, which limits direct translational conclusions. Future in vivo and pharmacokinetic studies are essential to validate biodistribution, circulation time, tumor-targeting efficiency, and overall therapeutic potential. Such investigations will provide a clearer understanding of the system’s clinical applicability and guide the development of nanoparticle-based therapies for liver cancer.

## Data Availability

The data supporting the findings of this study are available within the article. Raw data are available from the corresponding author upon reasonable request.
